# Efficacy of the current investigational drugs for the treatment of COVID-19: a scoping review

**DOI:** 10.1080/07853890.2021.1875500

**Published:** 2021-03-12

**Authors:** Ahmed Wadaa-Allah, Marwa S. Emhamed, Mohammed A. Sadeq, Nesrine Ben Hadj Dahman, Irfan Ullah, Nesrine S. Farrag, Ahmed Negida

**Affiliations:** a Biochemistry Department, Faculty of Science, Ain Shams University, Cairo, Egypt; b Faculty of Medicine, Tripoli University, Tripoli, Libya; c Faculty of Medicine, Misr University, Cairo, Egypt; d Faculty of Medicine of Tunis, University of Tunis El Manar, Tunis, Tunisia; e Kabir Medical College, Gandhara University, Peshawar, Pakistan; f Community Medicine Department, Faculty of Medicine, Mansoura University, Mansoura, Egypt; g Zagazig University Hospitals, Zagazig University, El-Sharkia, Egypt

**Keywords:** Coronavirus, drugs, treatment, SARS-COV-2, COVID-19

## Abstract

To date, there is no final FDA-approved treatment for COVID-19. There are thousands of studies published on the available treatments for COVID-19 virus in the past year. Therefore, it is crucial to synthesize and summarize the evidence from published studies on the safety and efficacy of experimental treatments of COVID-19. We conducted a systematic literature search of MEDLINE, PubMed, Cochrane Library, GHL, OpenGrey, ICTRP, and ClinicalTrials.gov databases through April 2020. We obtained 2699 studies from the initial literature search. Of them, we included 28 eligible studies that met our eligibility criteria. The sample size of the included studies is 2079 individuals. We extracted and pooled the available data and conducted a quality assessment for the eligible studies. From the 28 studies, only 13 studies provide strong evidence. Our results showed that Favipiravir and Hydroxycholoroquine shorten viral clearance and clinical recovery time and promote pneumonia absorption. On the other hand, Lopinavir-ritonavir either alone or combined with arbidol or interferons has no significant difference superior to the standard care. Corticosteroids, Convalescent plasma transfusion, and anticoagulant therapies provide a better prognosis. Remedsivir, Tocilizumab, Immunoglobulin, Mesenchymal stem cell transplantation showed effective treatment results, but further confirmatory studies are needed. In conclusion, Favipiravir and Remedsivir might be promising drugs in the treatment of COVID-19 patients.

## 1. Introduction

The novel coronavirus pandemic has spread from Wuhan, China, to over 200 countries and territories worldwide; worldometer statistics showed approximately 54.3 million confirmed cases, 1.3 million deaths and 37.9 million recovered patients. The clinical presentation of patients infected by the COVID-19 ranged from asymptomatic to critically ill cases requiring intensive care [1,2]. The significant causes of morbidity and mortality during hospitalization are acute respiratory distress syndrome (ARDS), arrhythmia and shock [3].

The current management and treatment are made on a case-by-case basis. The decision process includes evaluation of the severity of the clinical presentation, the feasibility of self-isolation, and the possibility of disease progression, which requires hospitalization for management [4]. Thus, there is an unmet clinical need to find safe and effective treatments to (1) manage the disease symptoms, (2) decrease ‘the viral load, (3) speed up recovery and therefore, (4) limit the viral transmission in the community [5].

As the COVID-19 clinical treatment is at a decisive point, it is imperative to synthesize all existing evidence available to determine if the existing evidence supports the current proposed management strategies. Thus, hundreds of clinical guidelines were published by local panels of each country using a methodologically rigorous process to evaluate the best evidence and provide treatment recommendations [6].

Until the moment, there are no FDA-approved treatments for COVID-19. However, the World Health Organization's International Clinical Trials Registry Platform declared that over 590 clinical trials are currently testing several potential treatments for COVID-19 [7]. These trials investigate: (1) pharmacological interventions; (2) advanced therapy medicinal products: cellular therapies, tissue extracts, plasma and vaccines for treatment; (3) non-pharmacological interventions: nutritional supplements and enteral feeds, physiotherapy and exercise, physical therapy, and psychotherapy [8].

Due to the lack of treatment approaches for COVID-19 as well as the urgent need to respond to the high morbidity and mortality rates caused by the COVID-19 pandemic, various studies were published including interesting approaches, the use of mesenchymal stem cells (MSCs) transplantation, tissue plasminogen activator (tPA), convalescent plasma, pharmacological intervention, without efficient peer-reviewing. Therefore, we performed this scoping review to assess and discuss different lines of treatment of COVID-19 for all ages and to summarize evidence from published studies about the safety and efficacy of these investigational treatments.

## 2. Methods

### 2.1. Literature search strategy

We conducted a systematic literature search of MEDLINE, PubMed, Cochrane Library, GHL, OpenGrey, ICTRP and ClinicalTrials.gov databases through April 2020 using the following search strategy: “(2019-nCoV OR 2019nCoV OR COVID-19 OR COVID-19 OR ((Wuhan AND coronavirus) OR Coronavirus Disease 2019) AND (Therapy OR Treatments)”.

### 2.2. Inclusion criteria

We included the intervention studies that met the following criteria: (1) preliminary and clinical trials as well as case series and any observational studies that report the efficacy of any treatment against COVID-19; (2) studies that report significant outcomes as clinical improvement outcomes, recovery rate, length of stay, discharge rate and mortality rate; (3) studies written in any language. On the other hand, we excluded the following studies: (1) studies with no sufficient efficacy endpoints; (2) case reports, conference abstracts, thesis, review articles, editorials, letter to editor and duplicate studies.

### 2.3. Study selection

We performed title and abstract screening after that we conducted a full-text screening for eligibility. Any disagreement was resolved by discussion with the two authors (Farrag N and Wadaa-Allah A).

### 2.4. Data extraction

We extracted the data independently on an excel sheet. The following data were extracted: (1) study design characterization (study design type, population, sample size and primary outcomes); (2) the baseline characteristics of the included studies (location, group, sample size, age, gender, intervention, treatment duration and primary clinical diagnosis). Any disagreement was resolved by discussion with authors (Farrag N and Wadaa-Allah A).

### 2.5. Quality assessment

According to the National Heart, Lung, and Blood Institute of the National Institutes of Health (NIH), we assessed the selected studies for quality assessment of controlled interventions, observational cohort and case series [9,10] (Table 1). Any disagreement was resolved by discussion with authors (Nesrine F and Wadaa-Allah A.).

**Table 1.** Quality assessment of the included studies.

Study ID	Score	Rate
Cao et al. [13]	8	Good
Tang et al. [14]	8	Good
Chen et al. [16]	9	Good
Cai et al. [18]	8	Good
Gautret et al. [5]	6	Good
Chen et al. [17]	10	Good
Runan et al. [15]	4	Poor
Leng et al. [11]	6	Poor
Ye et al. [12]	3	Poor
Jun et al. [19]	9	Good
Tang et al. [22]	8	Good
Jun et al. [21]	6	Good
Zha et al. [20]	8	Good
Wang et al. [28]	6	Good
Grein et al. [26]	7	Fair
Gautret et al. [27]	7	Good
Liu et al. [2]	4	Poor
Wang et al. [1]	6	Good
Dan et al. [33]	2	Poor
Shi et al. [34]	5	Fair
Luo et al. [31]	6	Poor
Zheng et al. [23]	4	Poor
Duan et al. [25]	4	Poor
Sun et al. [24]	7	Fair
Zhang et al. [29]	5	Poor
Shen et al. [30]	7	Fair
Chen et al. [32]	7	Fair
Wang et al. [35]	4	Poor

## 3. Results

### 3.1. Search results

**Figure 1.**
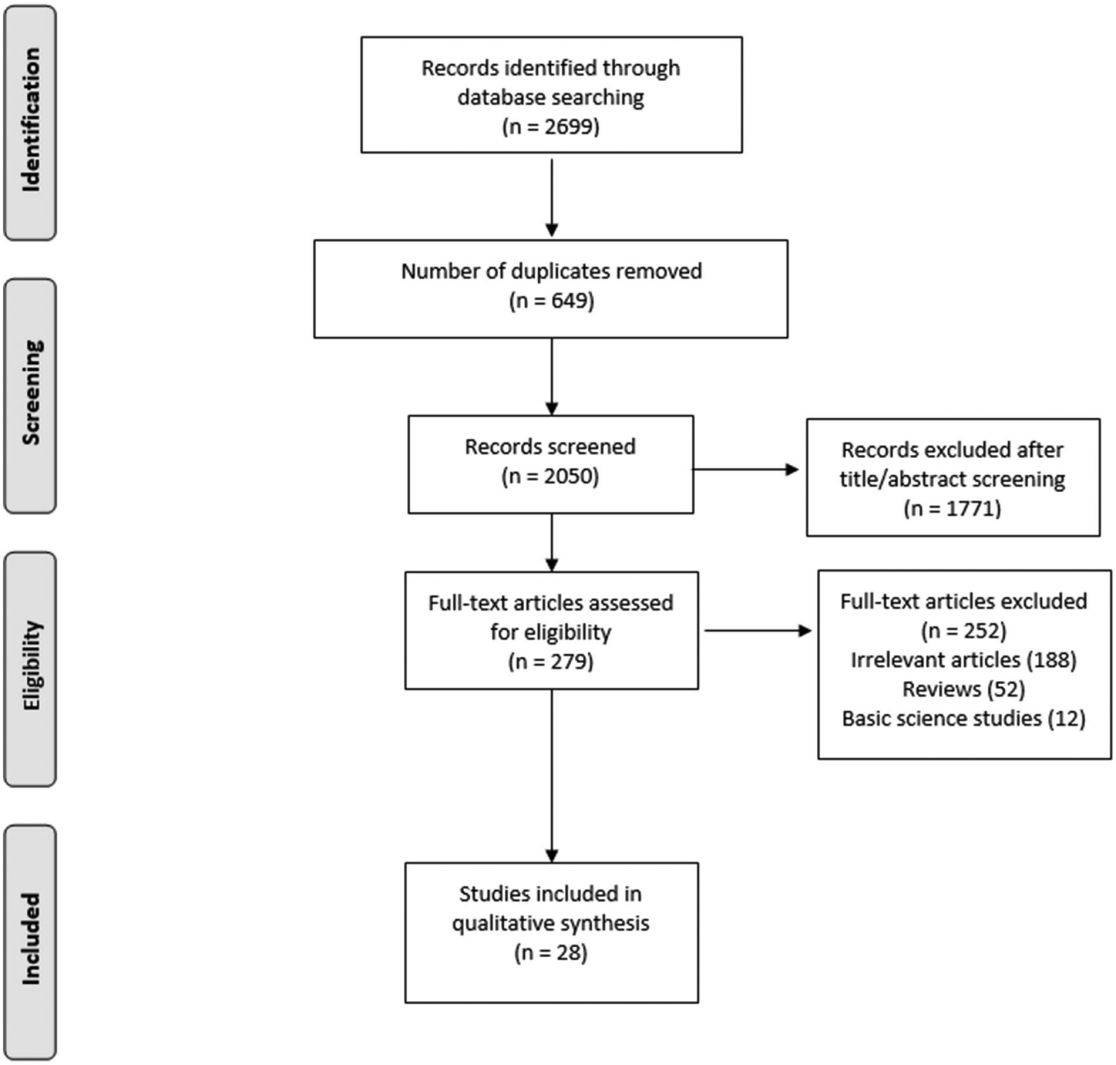
Flowchart describing the literature search process.

We found a total of 2699 studies in the initial literature search. Of them, 2671 papers were excluded because they did not meet our inclusion criteria. Eventually, 28 studies were included in our scoping review, 10 clinical trials [5,11–19], three retrospective cohort studies [20–22] and 15 case series studies [1,2,23–35]. Figure 1 summarizes the search process.

The treatments approached in these papers included hydroxychloroquine (HCQ), convalescent plasma transfusion, antiviral drug (lopinavir/ritonavir), corticosteroids, traditional Chinese medicine, anticoagulant therapy in patients with coagulopathy, lung transplantation and combined/non-specific treatments. We presented the baseline and summary of the included studies in Tables 2 and 3, respectively.

**Table 2.** Summary of the included studies.

Study ID	Study design	Population	Intervention	Sample size	Duration of treatment	Main findings
Cao et al. [13]	Randomized open-label clinical trial	Patients with severe COVID-19 infection	Lopinavir–ritonavir with standard care against standard care alone	199	28 d	Lopinavir–ritonavir treatment was stopped early in 13 patients (13.8%) because of adverse events. In hospitalized adult patients with severe COVID-19, no benefit was observed with lopinavir–ritonavir treatment beyond standard care
Tang et al. [14]	Randomized open-label clinical trial	Patients with COVID-19 infection	Hydroxychloroquine With standard care	150	28 d	The administration of hydroxychloroquine did not result in a higher negative conversion rate but it results in more alle*via*tion of clinical symptoms of the patients
Chen et al. [15]	Randomized open-label clinical trial	Patients infected with COVID-19	Hydroxychloroquine and standard treatment	62	24 d	Hydroxychloroquine could significantly shorten time to clinical recovery and promote the absorption of pneumonia
Cai et al. [18]	Randomized open-label clinical trial	Patients infected with COVID-19	Favipiravir against Lopinavir and ritonavir with IFN-α by aerosol inhalation	80	15 d	Favipiravir showed a better treatment effects in terms of disease progression and viral clearance than lopinavir and ritonavir
Gautret et al. [5]	Non-randomized open-label clinical trial	Patients with COVID-19 infection	Hydroxychloroquine and azithromycin, was added to treatment depending on their clinical presentation	36	16 d	Hydroxychloroquine treatment is significantly associated with viral load reduction/disappearance and its effect is reinforced by azithromycin
Chen et al. [17]	Pilot clinical trial	Patients with COVID-19 infection	Hydroxychloroquine sulphate treatment against control group	60	7 d	Moderate patients prognosis was good and future studies a better endpoint
Runan et al. [15]	Pilot clinical trial	Patients COVID-19 pneumonia	Triple treatment group: interferon a-2b, arbidol and, lopinavir/ritonavir against double treatment group: interferon a-2b and lopinavir/ritonavir	237	24 d	The earlier the three-drug combination antiviral treatment time, the faster the viral nucleic acid negative time
Leng et al. [11]	Pilot clinical trial	Patients with COVID-19 infection with no improvement signs were observed under the standard treatments.	Mesenchymal stem cells (MSCs) transplantation against placebo	10	25 d	The pulmonary function and symptoms of all patients were significantly improved in two days after MSC transplantation. Treatment was safe and effective
Ye et al. [12]	Pilot clinical trial	Patients with COVID-19 infection.	Lopinavir/ritonavir with adjuvant drugs against control patients that received adjuvant drugs only	47	14 d	Combination of LPV/ritonavir and adjuvant drugs has a more evident therapeutic effect than pneumonia-associated adjuvant drugs alone
Jun et al. [19]	Pilot clinical trial	Patients with common COVID-19 infection	Hydroxychloroquine sulphate along with standard treatment	30	20 d	It is difficult to determine the effect of the treatment plan in the study of the main endpoint of the virus conversion rate and the critical rate
Tang et al. [20]	Cohort study (retrospective)	Patients with coagulopathy combined with severe COVID-19 infection	Anticoagulant therapy using low molecular weight heparin	449	7–28 d	Anticoagulant therapy mainly with low molecular weight heparin appears to be associated with better prognosis in severe COVID-19 patients meeting SIC criteria or with markedly elevated D-dimer
Jun et al. [21]	Cohort study (retrospective)	Patients with COVID-19 infection	First group: interferon α2b, lopinavir and ritonavir against Second group that received interferon α2b and abidor, third group: interferon α2b and any antiviral drugs	134	17 d	It has not been found that lopinavir ritonavir and abidor have the effect of improving symptoms or shortening the negative time of viral nucleic acid in respiratory specimens
Zha et al. [20]	Cohort study (retrospective)	Patients infected with the acute COVID-19 infection	Corticosteroid with standard care	31	26 d	No association between corticosteroid therapy and outcomes in patients without acute respiratory distress syndrome
Wang et al. [28]	Case series (retrospective)	Patients, two mild and two severe COVID-19 pneumonia	Lopinavir/ritonavir (Kaletra^®^), arbidol and Shufeng Jiedu Capsule (SFJDC), a traditional Chinese medicine	4	6–15 d	All patients were improved. The efficacy of antiviral treatment including lopinavir/ritonavir, arbidol and SFJDC warrants further verification in future study
Grein et al. [26]	Case series (retrospective)	Patients with COVID-19	Remdesivir, 200 mg on day one then 100 mg daily in the following nine days	61	Median 18 d	Randomized clinical trials are required to measure the efficacy of remdesivir
Gautret et al. [27]	Case series (retrospective)	Patients with COVID-19 infection	Hydroxychloroquine and azithromycin combination	80	6–17 d	Hydroxychloroquine associated with azithromycin is effective in the treatment of COVID-19 infection
Liu et al. [2]	Case series (retrospective)	Patients with COVID-19 infection	Immunoglobulin G, corticosteroids with standard care	137	57 d	Systemic corticosteroid treatment did not show significant benefits
Wang et al. [1]	Case series (retrospective)	Patients with COVID-19 pneumonia	Arbidol with standard care	69	14 d	Arbidol treatment showed tendency to improve the discharging rate and decrease the mortality rate
Dan et al. [33]	Case series (retrospective)	Patients mild and severe COVID-19 pneumonia	Respiratory support, lopinavir, ritonavir, and abidor, moxifloxacin, cefoperazone, linezolid, imipenem, cilastatin, moxifloxacin, glucocorticoids	30	–	Most of the patients were improved
Shi et al. [34]	Case series (retrospective)	Patients with mild or severe COVID-19 pneumonia	Abidor, peramivir, ribavirin, oseltamivir phosphate, glucocorticoid therapy, immunoglobulin and albumin therapy	109	–	Treatment can improve prognosis and reduce mortality
Luo et al. [31]	Case series (retrospective)	Patients with moderate and severe COVID-19 pneumonia	Tocilizumab therapy	15	39 d	Tocilizumab appears to be an effective treatment option in COVID-19 patients with a risk of cytokine storms. For critically ill patients with elevated IL-6, repeated dose of the TCZ is recommended
Zheng et al. [23]	Case series (retrospective)	Paediatric patients with COVID-19 infection	Interferon, oseltamivir, cefoperazone/sulbactam, interferon, mechanical ventilation, systematic corticosteroids and intravenous immunoglobulin	25	–	The symptoms on admission were alle*via*ted 96% of the patients
Duan et al. [25]	Case series (retrospective)	Paediatric patients with mild/asymptomatic COVID-19 pneumonia	Interferon, oseltamivir, ribavirin, lopinavir/ritonavir, gamma globulin antibiotics, Chinese medicine	31	–	Given general treatment, the overall prognosis is good in the near future
Sun et al. [24]	Case series (retrospective)	Severe or critically ill paediatric patients with COVID-19 pneumonia	Symptomatic treatment and respiratory support, (virazole, oseltamivir and interferon) to all patients. Depending on patients’ conditions, intravenous glucocorticoids and immunoglobulin, traditional Chinese medicine was given	8	32 d	Most of the patients were improved and discharged
Zhang et al. [29]	Case series (prospective)	Critically ill patients with COVID-19	Convalescent plasma transfusion with standard care	4	18–46 d	Plasma reduce the relative risk of mortality of patients
Shen et al. [30]	Case series (prospective)	Critically ill patients of COVID 19 infection with ARDS	Convalescent Plasma transfusion with standard care	5	10–22 d	Convalescent plasma containing neutralizing antibody was followed by improvement in the clinical status
Chen et al. [32]	Case series (prospective)	Patients with pulmonary fibrosis related to acute respiratory distress syndrome due to COVID-19	Lung transplantation	3	30 d	Lung transplantation can be performed in end-stage patients with respiratory failure due to COVID-19-related pulmonary fibrosis
Wang et al. [35]	Case series (prospective)	Patients with COVID-19 infection	Tissue plasminogen activator (tPA) treatment with standard care	3	–	The observed improvements were transient and lost over time after completion of their tPA infusion. Further studies needed to confirm the results

SFJDC: Shufeng Jiedu Capsule; TCZ: tocilizumab; tPA: tissue plasminogen activator; ARD: acute respiratory distress syndrome.

**Table 3.** Baseline characteristics of the included studies.

Study ID	Location	Group	Sample size	Age Median (IQR) Mean ± SD Range	Gender Male (%)	Primary clinical diagnosis								
						Fever %	Cough%	Dyspnoea	WBC (10^9^/L)	Lymph (10^9^/L)	CRP (mg/dl)	IL-6 (ng/L)	Ct values	Chest CT score
Cao et al. [13]	China	Treatment	99	58.0 (50.0–68.0)	61.6%	89%	–	−	7.3 (5.3–9.6)	0.8 (0.6–1.4)	–	–	–	–
		Control	100	58.0 (48.0–68.0)	59.0%	93%	–	–	6.9 (4.9–9.1)	0.9 (0.5–1.2)	–	–	–	–
Tang et al. [14]	China	Treatment	75	48.0 ± 14.1	56.0%	59.70%	51.50%	22.10%	5.59 ± 1.90	1.46 ± 0.60	9.9 ± 13.30	12.90 ± 36.30	–	–
		Control	75	44.1 ± 15.0	53.3%	53.30%	38.20%	5.90%	5.60 ± 1.80	1.60 ± 0.50	7.4 ± 12.80	8.90 ± 13.00	–	–
Chen et al. [16]	China	Treatment	31	44.1 ± 16.1	45.2%	70.90%	70.10%		–	–	–	–	–	–
		Control	31	45.2 ± 14.7	48.3%	54.80%	48.40%	–	–	–	–	–	–	–
Cai et al [18]	China	Treatment	35	43 (35.5–59)	40.0%	62.9%	34.3%	–	8.1 (3.8–6.6)	1.5 (1.0–1.8)	15.0 (3.0–19.2)	14.0 (3.5–11.0)	30.7 (21.2–37.0)	12(4.0–14.0)
		Control	45	49 (36–61)	46.7%	82.2%	22.2%	–	4.3 (3.4–4.9)	1.2 (0.9–1.4)	21.4 (5.0–23.2)	12.9 (5.3–16.8)	29 (18.8–38.0)	10 (4.5–13.5)
Gautret et al. [5]	France	Treatment	20	51.2 ± 18.7	45.0%	–	–	–	–	–	–	–	–	–
		Control	16	37.3 ± 24.0	37.5%	–	–	–	–	–	–	–	–	–
Chen et al. [17]	China	Treatment	30	50.5 ± 3.8	60.0%	60.0%	–	–	5.20 (3.90–6.70)	1.11 ± 0.43	–	–	–	–
		Control	30	46.7 ± 3.6	40.0%	86.70%	–	−	4.90 (4.50–7.40)	1.18 ± 0.55	–	–	–	–
Runan et al. [15]	China	Treatment	196	45.7 ± 17	53.1%	–	–	−	5.1 (4.1–6.4.0)	−	11.8 (3.1–25.8)	–	–	–
		Control	41	45.1 ± 16.7	58.5%	–	−	4.2 (3.2–7.0)	−	8.1 (2.9–23.0)	–	–	–	–
Leng et al. [11]	China	Treatment	7	57.0 ± 7.70	57.1%	85.7%	71.5	71.4%	−	−	−	−	−	−
		Control	3	65.0 ± 16.47	0 %	33.3%	33.3	66.7%	−	−	−	−	−	−
Ye et al. [12]	China	Treatment	42	–	50.0 %	−	−	−	−	−	−	−	−	−
		Control	5	–	20.0%	−	−	−	−	−	−	−	−	−
Jun et al. [19]	China	Treatment	15	50.5 ± 3.8	60%	60%	–	−	1.11 ± 0.43	−	−	−	−	−
		Control	15	46.7 ± 3.6	80%	86.7%	–	−	1.18 ± 0.55	−	−	−	−	−
Tang et al. [22]	China	Survivors	315	63.7 ± 12.2	78.0%	−	−	−	−	−	−	−	−	−
		Non-survivors	134	68.7 ± 11.4	48.9%	−	−	−	−	−	−	−	−	−
Jun et al. [21]	China	Treatment	52	47 (35–60)	51.9%	92.3%	19.2%	1.9%	5.0 (3.7–5.66)	1.24 (0.93–1.56)	9.5 (0–26.8)	−	−	−
		Comparison	34	44 (34–62)	52.9%	91.1%	32.4%	0%	4.18 (3.44–5.19)	1.04 (0.72–1.33)	10.9 (4.0–25.2)	−	−	−
		control	48	55 (36–62)	50.0%	95.8%	22.9%	8.3%	5.09 (4.10–6.30)	1.23 (0.85–1.57)	19.5 (7.6 36.6)	−	−	−
Zha et al. [20]	China	Treatment	20	37 (27–52)	60%	70%	40%	5%	–	–	–	–	–	–
		Control	11	53 (36–57)	73%	100%	100%	27%	–	–	–	–	–	–
Wang et al. [28]	China	Treatment	4	44 (19–63)	75%	100%	75%	50%	−	6.17 (4.4–10.84)	–	–	–	50%
Grein et al. [26]	Data collected from different countries	Treatment	61	64 (48–71)	75%	–	–	–	–	–	–	–	–	–
Gautret et al. [27].	France	Treatment	80	52.5 (42–62)	52.5	15%	58.8%	–	–	–	−	–	23.6 ± 4.3	–
Liu et al. [2]	China	Treatment	137	57 (20–83)	44.5%	48.3%	48.2%	19%	43.8%	27.7%	83.9%	–	–	–
Wang et al. [1]	China	Treatment	69	42 (35–62)	46%	44.0%	55.0%	29.0%	3.82 (2.98–5.57)	1.15 (0.82–1.46)	13.2 (6.78–49)	8.54 (4.68–20.58)	9.0%	–
Dan et al. [33]	China	Treatment	30	43 (21–72)	60%	–	–	–	–	–	–	–	–	–
Shi et al. [34]	China	Treatment	109	52.5 ± 10.8	44%	95.5%	86.5%	21.3%	−	53.2%	79.8%	−	24.8%	–
Luo et al. [31]	China	Treatment	15	73 (62–80)	80%	–	–	–	–	–	–	–	–	–
Zheng et al. [23]	China	Treatment	25	3 (2–9)	56%	52.0%	44.0%	8.0%	6.2 (4.30–9.85)	2.19 (1.15–3.31)	14.5 (0.93–25.04)	−	68.0%	–
Duan et al. [25]	China	Treatment	31	7 (0.5–17)	–	65%	45%	–	−	6%	10%	–	–	–
Sun et al. [24]	China	Treatment	8	3 (0.6–15)	75%	75%	75%	–	−	8.10 (1.5–14.95)	25.8 (0.5 − 103)	10.07 (63.99–2.92)	–	–
Zhang et al. [29]	China	Treatment	4	57 (31–73)	50%	–	–	–	–	–	–	–	–	–
Shen et al. [30]	China	Treatment	5	54 (36–73)	60%	80%	–	–	–	−	130.62 (65–242.8)	−	25.52 (18.9–38.0)	–
Chen et al. [32]	China	Treatment	3	66 (58–73)	100%	–	–	−	–	0.56 (0.41–0.7)	–	–	–	–
Wang et al. [35]	China	Treatment	3	61 (49–75)	67%	100%	100%	67%	–	–	–	–	–	–

IQR: interquartile range; SD: standard range; WBCs: white blood cells; lymph: lymphocytes; CRP: C-reactive protein; IL-6: interleukin-6; CT: computed tomography.

#### 3.1.1. Hydroxychloroquine

Jun et al. performed a pilot clinical trial on 30 cases with common COVID-19 infection, mean age ± SD (50.5 ± 3.8, 46.7 ± 3.6) for the treatment group, and control group, respectively. The treatment group received HCQ sulphate (400 mg, once per day for five days) plus standard treatment while the control group received only the standard care which is bed rest, oxygen inhalation, antiviral drugs as alpha-interferon nebulization, oral lopinavir/ritonavir and antibiotics (if recommended). The drug was ceased for one patient that became a severe case. 86.7% of the treatment group and 93.3% had negative viral pharyngeal swabs on the seventh day. For radiological imaging, 33.3% of the treatment group and 46.7% in the control group improved on day three of enrolment. Following re-examination, the lesions were improved in all patients. The study concluded that a larger sample size study is needed to investigate the effects of HCQ in the treatment of COVID-19 and it is difficult to determine the effect of the treatment plan on the main endpoint of the virus conversion rate and the critical rate [17].

In an open-label non-randomized clinical trial 42 patients above 14 years of age and RT-PCR, which is the most powerful tool in virus detection [36,37], positive in patients with nasopharyngeal swab were enrolled in the study. Of the 42 patients who met the inclusion criteria, six missed follow-up and 36 were enrolled in the study. Out of the 36, 16 were group controlled, and 20 were a group of HCQ-treated patients. Of 36 patients, 15 were male (41.7%), averaging 45.1 years of age. The proportion of asymptomatic patients was 16.7%, 61.1% of patients with symptoms of upper respiratory tract infection, and 22.2% of patients with symptoms of lower respiratory tract infection. All patients with symptoms of lower respiratory tract infection had confirmed pneumonia on a computerized tomography (CT) scan. Patients treated with HCQ were older than those with control (51.2 years vs. 37.3 years). At post-inclusion day 6, 70% of patients treated with HCQ were recovered compared to 12.5% in the control group (*p*=.001). At day 6 post-inclusion, 100% of patients treated with a combination of HCQ and azithromycin were recovered compared to 57.1% of patients treated with HCQ alone and 12.5% in the control group (*p*<.001). By comparison, at day eight of postinclusion, one patient under a combination of HCQ and azithromycin, who tested negative on day six post inclusion, was tested positive at low titre. Thus, the treatment of hydroxy chloroquine in COVID-19 patients is significantly correlated with viral load reduction/disappearance, and its effect is enhanced by azithromycin [5].

A recent randomized clinical trial on 62 patients with mean age ± SD (44.7 ± 15.3) patients in the treatment group received standard care with HCQ in addition to HCQ sulphate and the control group received only standard care. For time to clinical recovery, the body temperature recovery time and the cough remission time were significantly shortened in the hydroxy treatment group. Additionally, 80.6% of the treatment group showed a significant clinical improvement of pneumonia compared with the control group (54.8%). Thus, HCQ could significantly shorten time to clinical recovery and promote the absorption of pneumonia [16].

Gautret et al. conducted a retrospective data analysis on 80 patients with COVID-19 infection with age range (18–88 years) who received HCQ in combination with azithromycin for at least three days and a follow-up period of at least six days. Their results showed that 81.3% of the patients had improved outcomes, qPCR test was negative in 93% of the total patients on day eight, and was discharged, 15% required oxygen therapy, 3.75% were transferred to the ICU and finally one patient dead. This study showed that HCQ associated with azithromycin is effective in the treatment of COVID-19 infection [27].

A larger open-label randomized clinical trial conducted on 150 patients with COVID-19, mean age ± SD (46.1 ± 14.7 years). The patients were assigned in two groups, an experimental group which received HCQ as long as standard care (provision of intravenous fluids, supplemental oxygen, normal laboratory testing, and COVID-19 test, hemodynamic monitoring and intensive care and the potential to supply concomitant medications) and a control group received standard care only. Hydroxychloroquine is efficient to ease the symptoms when confounding factors of anti-viral agents were removed in the post hoc analysis. Furthermore, CRP in the control group in much greater than the treatment group (6.986 in standard care and HCQ group vs. 2.723 in the control group, mg/L). Besides, there is a rapid recovery of lymphopenia. Adverse events were found in 8.8% of control individuals and 30% of HCQ recipients with two serious adverse events. The most common adverse event in the treatment group was diarrhoea (10%). Furthermore, the administration of HCQ did not result in a higher negative conversion rate but more alleviation of clinical symptoms than standard care only alone in patients hospitalized with COVID-19 without receiving antiviral treatment, possibly through anti-inflammatory effects [14].

#### 3.1.2. Convalescent plasma

In a case serious study that included critically ill patients with a laboratory-confirmed COVID-19 (age 36–65 years), out of them, two patients were women. Inclusion criteria were severe pneumonia with rapid progression, high viral load despite the use of antiviral and mechanical ventilation. They received mechanical ventilation at the time of treatment, and all participants received antiviral agents and methylprednisolone. All patients received transfused with convalescent plasma. In four out of five patients following plasma transfusion, temperature normalized. Viral loads also decreased and were negative within 12 days of transfusion, and following transfusion, COVID-19-specific ELISA and neutralizing antibody titres increased (range, 40–60 before and 80–320 on day seven). ARDS was resolved 12 days after transfusion in four patients, and three patients were removed from mechanical ventilation within two weeks of treatment. Of the five patients, three have been discharged from the hospital, and two are in stable condition at 37 days after transfusion. Thus, the convalescent plasma administration improved the clinical outcome of critically ill patients with COVID-19 and ARDS [30].

Also, in another case series study of four cases of critically ill patients with COVID-19. Three of them developed severe ARDS despite being treated by arbidol, lopinavir–ritonavir, interferon-alpha inhalation and other supportive therapies. One case experienced septic shock, and she was given a whole of 900 mL O-compatible convalescent plasma was transfused to the patient in three batches; the first batch was given at 8 AM on the 17th of February (200 mL), the 2nd one was at 8 AM on the 27th of February (400 mL) and the closing one was at 8 AM on the 28th of February (300 mL). The virus load of the patient on the 18th of February was 55 × 10^5^ copies/mL, which appreciably reduced to 3.9 × 10^4^ copies/mL on the 28th of February, and besides reduced to 180 copies/mL on 5th of March. The patient was extubated on 3rd March, CT scan shows persistent absorption of consolidation. RT-PCR of oropharyngeal swab done on 9th and 10th of March were negative for COVID-19. The patient was discharged on the 13th of March. Another case developed ARDS and was given non-invasive mechanical ventilation. Follow up CT scan shows interstitial pneumonia extended to both lungs. When 200 mL convalescent plasma from a COVID-19 recovered patient was transfused to this patient, no adverse effect was observed, and patient RT-PCR came negative and was discharged. The third case was found to have multi-organ failure and develop septic shock. Eight transfusions of B-compatible convalescent plasma (2400 mL) have been given to the patient. The patient viral load decreased and two RT-PCR of sputum in deep lung came negative. The patient was transferred to an unfenced ICU for the management of underlying diseases and a couple of organ failure. The fourth case was once a 31-year-old pregnant woman. CT scan of the chest confirmed opacities in the lower lobe of the left lung. The affected person developed severe ARDS, multiple organ dysfunction syndromes, and septic shock after admission. The affected person was in a ventilated and caesarean section done; however, due to endouterine asphyxia newborn died. Cardiac ultrasound counselled left ventricular enlargement with decreased systolic function. The affected person obtained invasive ventilation and continuous renal replacement therapy: treatment with lopinavir–ritonavir (400 mg twice daily) and ribavirin (500 mg every 12 h). Three hundred millilitres transfusion of convalescent plasma was once given to the patient. The outcomes of two continual RT-PCR assessments of BAL fluid got here were negative. The patient recovered from COVID-19 and was discharged. Convalescent plasma would possibly be a therapy for critically ill patients infected with COVID-19 and no unfavourable effect was observed [29].

#### 3.1.3. Tocilizumab treatment

Luo et al. conducted a retrospective study on 15 patients with moderate and severe COVID-19 pneumonia with age range (62–80 years). Totally, 15 patients with COVID-19 were included in this study. The patients received tocilizumab (TCZ) ranging from 80 mg to 600 mg per day for one to seven days. Two of the patients were moderately ill, six were severely ill and seven were critically ill. Tocilizumab was used in combination with methylprednisolone in eight patients. Five patients received the TCZ administration twice or more. Tocilizumab decreased the CRP in all patients rapidly, but for the four critically ill patients who received only a single dose of TCZ, three of them dead and the CRP level in the remaining patient did not decrease to the normal range with a clinical outcome of disease aggravation. Serum IL-6 decreased after TCZ therapy in 10 patients and did not decrease in patients not affected by the treatment. A persistent and dramatic increase of IL-6 was observed in these four. In COVID19 patients at risk of cytokine storms 21, TCZ appears to be an appropriate treatment choice [31].

#### 3.1.4. Corticosteroids

A retrospective cohort study of 31 patients diagnosed with coronavirus was performed in two hospitals. Their median age was 39 (IQR, 32–54 years); 20 (64%) were men. Seven patients had hypertension, two had chronic hepatitis B virus (HBV) infection (virus loads, 2950 copies, and 3040 copies/mL; both patients had entecavir), one had diabetes and one had coronary heart disease. Two patients had a smoking history. No patients reported chronic respiratory diseases, cancer or other chronic diseases. Twenty-nine patients’ CT shows pneumonia finding in 20 patients’ bilateral involvement while in two patients no CT finding was present. The outcomes were viral clearance time, hospital stay and symptom duration. Eleven out of 31 were given corticosteroid (methylprednisolone 40 mg once or twice a day) within 24 h of admission. Corticosteroid-receiving patients had a higher median CRP level (*p*=.026) and a lower median lymphocyte count (*p*=.012) compared to non-corticosteroid patients [20]. Twenty-six (84%) of the 31 patients had recovered from COVID-19 and were discharged and five were still in the hospital. The median time to clear up the virus was 14 days (IQR, 12–16 days; range, 7–26 days). The median duration of symptoms was seven days (IQR, 5–10 days); the median length of hospital stay was 18.5 days (IQR, 16–21 days). There was no association found of steroid with hospital length of stay (HR, 0.77; 95% CI, 0.33–1.78), duration of a symptom (HR = 0.86; 95% CI, 0.40–1.83) and viral clearance time (HR = 1.26; 95% CI, 0.58–2.74). Eventually, there was no association found between corticosteroid use and outcome in patients without ARDS, and an existing HBV infection delay viral clearance [20].

A case series include 137 patients to describe the prognosis and clinical outcomes of non-specific COVID-19 treatment including antiviral treatment, antibacterial treatment, systemic corticosteroids, γ-immunoglobulin and respiratory support. Patients were recruited in nine tertiary hospitals in Hubei, China. Of the 137 patients included in this study; 105 (76.6%) received antiviral treatment; 119 (86.9%) received antibacterial treatment; 40 (29.2%) received systemic corticosteroids; 44 (32.1%) received human γ-immunoglobulin; and 119 (86.9%) received respiratory support with nasal cannula (85[62%]) and non-invasive ventilation (34[24.8%]). Regarding prognosis of these patients; 44 (32.1%) improved and were discharged; 77 (56.2%) inpatient treatment; and 16 (11.7%) died. They concluded that systemic corticosteroid treatment did not show significant benefits [2].

#### 3.1.5. Anticoagulant therapy

In a retrospective observational study to assess the role of heparin treatment in severe cases of COVID-19 with coagulopathy, 449 patients were included in the study, with a mean age of 65.1 ± 12.0 years. About 61% of these patients have comorbidities, mainly diabetes and hypertension, heart diseases. Anticoagulant treatment was defined as receiving heparin treatment for at least seven days. All patients received the standard treatment, including antiviral and supportive treatment. Twenty-two percent of the patients (*n* = 99, 94 received low molecular weight heparin (40–60 mg enoxaparin/day), and five received unfractionated heparin (10,000–15,000 U/day)). After 28 days of treatment, there was no statistical difference in the mortality rate between both groups heparin users and non-users (30.3% vs. 29% respectively, *p*=.9), but in stratified analysis, the mortality among heparin users was lower than nonusers. In patients with sepsis-induced coagulopathy (SIC) (SIC score) score ≥4 (40.0% vs. 64.2%, *p*= .029), or D-dimer >3.0 μg/mL (32.8% vs. 52.4%, *p*= .017). The study concluded that heparin treatment improves the prognosis of COVID-19 cases with coagulopathy [22].

Wang et al. performed a case series study on three patients with COVID-19 infection, age range (49–75 years). The patients were undergoing administration of tPA with standard care. The three patients showed an improvement in their P/F ratio ranging from 38% improvement to a ∼100%. The D-dimer decreased in two cases only and the fibrinogen levels increased in one case and remained similar in another and not reported in the remaining case. Thus, more studies are needed to confirm these results [35].

#### 3.1.6. ACE2-mesenchymal stem cells transplantation

A pilot trial was conducted in a hospital in Beijing on seven patients aged between 18 and 95 years who were RT-PCR-positive with intravenous MSCs transplantation. Seven confirmed COVID-19 patients were registered for MSCs transplants, including one critically severe type, four severe types, and two common types, and three severe types were enrolled for placebo control. MSCs were suspended in 100 mL of normal saline before the intravenous drip, and the total number of cells transplanted was calculated by 1 × 10^6^ cells per kilogram of weight. The window period for cell transplantation was described as the time when symptoms or/and signs deteriorated even as the expectant therapies were being performed. The injection was conducted with a speed of ∼40 drops per minute, for around 40 min. The patients were examined by the investigators after receiving the investigational drug via the 14-day observation. The clinical results and improvements in rates of inflammatory and immune function and adverse effects of seven enrolled patients were monitored for 14 days. In two days after MSCs transplantation, these seven patients' pulmonary function and symptoms were significantly improved. Two common and one severe patient were recovered and discharged within 10 days following treatment. After treatment, the peripheral lymphocytes increased, the C-reactive protein decreased, and the CXCR3-CD4-T cells, CXCR3 + CD8 + T cells and CXCR3 + NK cells disappeared within three to six days of treatment. Besides, a team of CD cell populations with regulatory CD14 + CD11c + CD11bmid increased dramatically. While levels of tumour necrosis factor alpha reduced significantly, IL-10 has risen in the MSCs treatment group as opposed to the placebo control group. The gene expression profile also showed that MSCs were ACE2- and TMPRSS2- which suggested that MSCs are free of COVID-19 infection. Intravenous transplantation of MSCs was safe and effective for treatment in COVID-19 pneumonia patients, particularly in critically severe patients [11].

#### 3.1.7. Antivirals

Jun et al. reviewed the records of 134 cases (aged from 35 to 62 years with an average of 48 years). Patients were divided into three groups; the first group included 52 patients and received lopinavir/ritonavir oral tablets two times a day for five days; the second group included 34 patients and received arbidol 200 mg three times a day for five days, and the third group included 48 patients and did not receive any antiviral drug as a control group. All patients in this study received recombinant human interferon a2b spray. A total of five patients had developed severe illness; two patients (3.8%) in the lopinavir group; one (3.3%) in the arbidor group; and two (4.5) of the control group, and there was no significant difference between three groups in time of having negative PCR test; the percentage of negative conversion in lopinavir group was 71.8%; in arbidor group was 82.6%, and control group was 77.1% (*p*= .79). They concluded that lopinavir–ritonavir and abidor had no effect of improving symptoms or shortening the negative time of viral nucleic acid in respiratory specimens [21].

Similarly, in the second study (randomized open-label clinical trial), 199 patients were recruited from 18 January 2020 to 3 February 2020, in Jin Yin-Tan Hospital. Patients were divided into two groups: the first group included 99 patients and received lopinavir (400 mg)/ritonavir (100 mg) oral tablets two times a day with a standard of care and the second group included 100 patients and received standard of care alone. Standard of care included supplemental oxygen either non-invasive or invasive according to need, antibiotics, vasopressors, renal replacement therapy and ECMO. There was also no significant difference between the lopinavir/ritonavir group and the control group in clinical improvement (median 16 days vs. 16 days, the hazard ratio for clinical improvement, 1.31; 95% CI, 0.95–1.85; *p*=.09). Also there was no significant difference between the lopinavir/ritonavir group and the control group in body temperature (median 36.5 °C IQR [36.4–37.0], 36.5 °C IQR [36.5–36.8]); WBCs (median 7.3 IQR [5.3–9.6]), 6.9 IQR [4.9–9.1]); or platelet count (median 201.0 IQR [155.0–287.0], 210.0 IQR [163.0–269.5]) [13].

Ye et al. study included 47 patients recruited from Rui’an People’s hospital. They divided their patients into two groups: the first group included 42 patients and received lopinavir/ritonavir plus standard adjuvant therapy. The second group included five patients and received standard adjuvant therapy alone. Adjuvant therapy included interferon aerosol inhalation, arbidol tablets, asmeton, eucalyptol limonene, pinene enteric soft capsules, moxifloxacin and supplemental oxygen as needed. The results have shown a faster return to normal body temperature in the lopinavir/ritonavir group compared to the control group (test group: 4.8 ± 1.94 days against control group 7.3 ± 1.53 days, *p*=.0364). It was also shown that the abnormal blood picture (WBCs, lymphocytes, CRP, PLT) was generally lower in the lopinavir/ritonavir group than the control group [12].

Regarding remdesivir, 61 patients received remdesivir but there were eight patients that were not analysed (seven with no post-treatment data and one has a dosing error). The remaining 53 patients were analysed, 75% received the full 10-day course of remdesivir, 19% received five to nine days of treatment and 6% fewer than five days of treatment. During a median follow-up of 18 days, 36 patients (68%) had an improvement in oxygen-support class, including 17 of 30 patients (57%) receiving mechanical ventilation who were extubated. A total of 25 patients (47%) were discharged, and 13% died; mortality was 18% (six of 34) among patients receiving invasive ventilation and 5% (one of 19) among those not receiving invasive ventilation. In conclusion, randomized clinical trials are required to measure the efficacy of remdesivir [26].

Furthermore, favipiravir showed significant improvement in chest CT of the experimental group compared with the control group, with an improvement rate of 91.43% vs. 62.22%. These results show that favipiravir improved the prognosis of COVID-19 patients [18].

#### 3.1.8. Combined/non-specific treatments

A multicentre clinical trial included 237 patients who were recruited in 15 medical centres in Zhejiang province in the period from 22 January 2020 to 16 February 2020. Patients were divided into two groups; the first group included 196 patients with a mean age of 45.7 ± 17 years; and the second group of 41 patients with a mean age of 45.1 ± 16.7 years. First group had 92 (46.9%) females and 104 (53.1%) males; and the second group contained 17 (41.5%) females and 24 (58.5%) males. Both groups had similar percentages of chronic diseases including hypertension (first group: 38 [19.4%], second group: nine [22.0%], *p*=.780); diabetes (first group: 15 [7.7%], second group: four [9.8%], *p*=.534); and chronic liver disease (first group: 11 [5.6%], and second group: five [12.2%], *p*=.236). Regarding assigned treatment, the first group was the triple antiviral therapy group, they received arbidol (200 mg, three times a day), lopinavir/ritonavir (two tablets two times a day) and recombinant interferon α-2b (five million U two times a day, inhalation) for five days. The second group received only lopinavir/ritonavir and recombinant interferon α-2b. No statistical difference was found between the two groups in median hospital stay (*Z* = 6.722, *p*>.05) [15].

In a retrospective case series to assess the outcome of patients receiving combined lopinavir/ritonavir (Kaletra^®^), arbidol and Shufeng Jiedu Capsule (SFJDC), a traditional Chinese medicine, four patients were recruited in Shanghai Public Health Clinical Center, Shanghai, China and diagnosed with COVID-19 according to World Health Organization (WHO) guidelines (two patients < 35 years, two patients >60 years). By the end of study, two of the four patients were discharged and the other two remained in the hospital. All the patients received oxygen therapy via nasal cannula, antibiotic treatment and antiviral treatment including Kaletra^®^ (lopinavir 400 mg/ritonavir 100 mg, every 12 h), arbidol (0.2 g three times a day) and SFJDC (2.08 g, three times a day). Antiviral treatment was continued for 6–15 days. All patients had improved, three patients were discharged. Antiviral therapy’s effectiveness includes lopinavir/ritonavir, arbidol and SFJDC, which needs further testing in future studies [28].

Another case series described the clinical outcomes of patients receiving antiviral therapy, antibiotic therapy, antifungal therapy, corticosteroids and arbidol. All patients in this study were consecutively recruited in a period from 16 January 2020 to 29 January 2020, at the Union hospital in Wuhan, China. The recruited 69 patients were confirmed to have COVID-19 using PCR tests of throat swabs. The patients had a median age of 42.2 (IQR 35.0–62.0); 32 (46%) were males and 37 (54%) were females. Two patients were excluded from the analysis of treatment because of the transfer. Twenty-nine percent of patients showed dyspnoea and 20% of cases showed SpO2 < 90%. As of 4 February 2020, 18 (26.9%) of 67 patients had been discharged, and five patients had died, with a mortality rate of 7.5%. The study concluded that arbidol treatment showed the tendency to improve the discharging rate and decrease the mortality rate [1].

Dan et al. reported a case series with 30 patients recruited from Sixth People's Hospital of Shenyang in the period from 22 January 2020 to 8 February 2020 (ages ranging from 21 to 72 and a median age of 43 years). Eight (27%) cases had chronic disease including atherosclerosis, hypertension, diabetes, cerebral infarction or bronchitis. Thirteen (43%) cases received oxygen supplementation; 30 (100%) cases received antivirals including lopnavir, ritonavir and arbidol; 12 (40%) received antibiotics including moxifloxacin, cefoperazone, linezolid and imipenem cilastatin; and eight (26.7%) patients received glucocorticoids. By the end of the study, nine patients were discharged, 20 improved, one unchanged severe disease and no deaths [33].

Another case series included 109 patients from Wuhan's sixth hospital during the period from 24 December 2020 to 28 January 2020 (mean age ± SD 52.5 ± 10.8). Comorbidities among the patients included; 20 (19.2%) smokers; and 39 (35.8% cases) with underlying chronic diseases including lung diseases, sexual diseases and cardiovascular diseases [40]. All patients received antiviral therapy: 38 (39.4%), received oseltamivir phosphate (75 mg oral twice a day), three cases (2.8%) received ribavirin (500 mg IV drip, one time/day), three cases (2.8%) received peramivir, five cases (4.6%) received arbidor tablets (0.2 oral three times a day) and 60 cases (55.5%) received a combination of two antivirals. Other participants received treatments that include antibiotics (cephalosporins, quinolones and macrolides according to need), 12 patients received antifungals, 19 patients received α-interferon, 58 patients (53.2%) received glucocorticoids, 68 cases (62.4%) received immunoglobulin and 98 (90.0%) received oxygen supplementation either non-invasive or high flow humidification. By the end of this study, 51 cases (46.8%) were discharged, 10 cases (9.2%) were transferred, seven cases (6.4%) died, 48 cases (44.0%) were still hospitalized, 26 cases improved [34].

#### 3.1.9. Treatment of paediatric patients

A retrospective observational study included 31 paediatric patients with mild/asymptomatic pneumonia. Twenty-nine cases (94%) of children received antiviral treatment, including 10 cases received interferon alone, one case received oseltamivir alone, the remaining patients received the combination of two or more drugs of interferon, oseltamivir phosphate, ribavirin, Abidor (oral, 6–16 days), lopinavir/ritonavir. Except for one patient who had a slight increase in serum transaminase, no other adverse reactions were seen. Six children were treated with antibacterial drugs. Two children were treated with intravenous infusion of gamma. Eight children received symptomatic Chinese medicine decoction oral treatment. One patient was not treated and the remaining received an intravenous infusion of Xiyanping and Yiqiyangyin decoction. All children were not given glucocorticoid and mechanical ventilation. The discharge rate was 77%. In brief, the overall prognosis was good soon by general treatment [25].

Another retrospective case series study was performed on 25 children with COVID-19 infection with an age median of three years and interquartile range from two to nine years. Forty-eight percent of the patients received antiviral therapy (interferon, arbidol, oseltamivir, lopinavir/ritonavir), 56% who received empirical antibiotics were treated with empirical antibiotics. One patient showed bacteriological efficacy following treatment. There were two critical cases additionally given invasive mechanical ventilation, systematic corticosteroids and intravenous immunoglobulin. One of them received kidney replacement therapy. One patient completely recovered and was discharged. The clinical symptoms were improved in 96% of the patients. The study concluded that children were susceptible to COVID-19 like adults, while the clinical presentations and outcomes were more favourable in children [23].

Sun et al. performed an observational study on eight severe or critically ill paediatric patients with COVID-19 pneumonia, median age two months to 15 years. All patients received antiviral treatments (virazole, oseltamivir and interferon). According to the patient’s condition, antibiotics, traditional Chinese medicine, intravenous glucocorticoids and immunoglobulin were used. The discharge rate was 62.5% [24].

#### 3.1.10. Lung transplantation

Lung transplantation for three patients with pulmonary fibrosis was related to ARDS due to COVID-19, age range (58–73 years). Lung transplantation is the unique therapy for end-stage pulmonary fibrosis as rescue therapy for these patients. Two of the three recipients survived after liver transplantation. Lung transplantation can be performed in end-stage patients with respiratory failure due to COVID-19-related pulmonary fibrosis [32].

#### 3.1.11. Summary of current COVID-19 treatments

The only evidence-based interventions that can decrease the mortality and morbidity of severe COVID-19 patients with ARDS are respiratory support, mechanical ventilation and ECMO. The therapies with plasma and antibodies obtained from convalescent patients have been proposed to treat severe COVID-19 cases. The efficacy of all available drugs is still questionable. Nonetheless, they are currently prescribed for COVID-19 patients as off-label and compassionate-use till the time well-designed, randomized controlled trials are published. A summary of the currently used COVID-19 treatments is shown in Table 4.

**Table 4. Summary of results about covid-19 available drugs.** 

Drug	Nature	Mode of action	Ref.
Hydroxy chloroquine	Antimalarial drug	Inhibit viral replication	[5,14,16,17,27]
Favipiravir	Inhibitor of RNA polymerase	Inhibit RNA polymerase to inhibit the replication of viral RdRp gene	[41]
Immunoglobulin	Inhibitor of Fc receptor	Neutralize endogenous antibody especially for enteroviruses	[42,43]
Arbidol or umifenovir	Antiviral drug	Inhibit the fusion of haemagglutinin membrane	[1,12,21,23,28,44,45]
IFN-b1b	Cytokine	Inhibit mRNA synthesis and its translation to proteins	[46]
Remdesivir	Adenosine analogue	Inhibit viral replication through inhibition of RNA polymerase	[26,47,48]
Baloxavir marboxil	Cap-dependent endonuclease inhibitor	Inhibit viral Cap-dependant endonuclease	[49]
Lopinavir/ritonavir	Protease inhibitor	Inhibit the activity of 3CL^PRO^ and PL^PRO^ proteases of the COVID-19 virus	[17,28,29,50–52]
Nafamostat/mesilate	Serine protease and TMPRSS2-inhibitor	Inhibit coagulation, complement and kallikrein–kinin systems	[6]
Camostat/mesilate	TMPRSS2-inhibitor	Inhibit fusion and replication of COVID-19 virus	[53]
Jakotinib hydrochloride	JAK inhibitor	Inhibit AAK1 and JAK	[54]
Ruxolitinib	JAK inhibitor	Inhibit protein tyrosine kinases, JAK inflammation and cellular proliferation	[55]
Baricitinib	JAK inhibitor	Lowering COVID-19 endocytosis and inhibit JAK1/2 and AAK1	[56]
Meplazumab	Humanized multiple antibodies	Inhibit the activity of IL-5	[57]
Tocilizumab	IL-6 inhibitor	Has a role in the inhibition of cytokine syndrome	[31]
Corticosteroids	Glucocorticosteroid hormones	Cease inflammatory and fibrotic reactions	[2,6]

## 4. Discussion

Most of the studies report the outcome of patients receiving combined treatment, including antiviral, corticosteroids, supportive treatments, respiratory support, interferon. These studies were urgently designed to assess the outcome of the treatment rather than following the guideline and describing patients' outcome.

Antimalarial drugs, chloroquine and HCQ have been anticipated as treatments that could reduce transmission of the virus. *In vitro* studies showed that these drugs can both inhibit COVID-19 transmission through alkalization of the intracellular phagolysosome, which hinders virion fusion and uncoating and, consequently, viral spread [38,39]. Early clinical studies in China propose that chloroquine use might be associated with delayed symptoms [40]. Hydroxychloroquine has several advantages, being not expensive, readily available, and a well-known safety profile. The evidence behind the beneficial effect of them is still weak. Four studies reported improvement of clinical symptoms [5,14,16,27], but one only of them reported that HCQ was significantly associated with viral load reduction/disappearance [5]. Gautret et al. elucidated that HCQ treatment is significantly associated with viral load reduction/disappearance and its effect is reinforced by azithromycin but the study has several limitations: a small sample (20 participants who received HCQ, and 16 controls), a short observation period (six days), and no randomization, which raises concerns about selection bias [5]. The other French study reported that the combination of HCQ and azithromycin was associated with reduced viral load, but no other clinically relevant outcomes were reported. The study was non-controlled, with poorly defined inclusion and exclusion criteria [27]. The other randomized Chinese study, which included larger cohorts, found that HCQ did not result in a higher seroconversion rate but more alleviation of clinical symptoms, possibly through anti-inflammatory effects. But this study was open labelled [14]. The clinical observations of the effects of HCQ for patients with COVID-19 have not involved critically ill patients who are receiving several other medications and may have organ failure, which affects drug metabolism and potentially increase the risk of adverse events [27,58].

Convalescent plasma (passive polyclonal antibody) improved patients' survival rate with viral causes [59]. Regarding its results in the treatment of COVID-19, there were two case series studies on a small number of patients [29,30]. Convalescent plasma leads to substantial improvement in patients' clinical status and reduces the mortality rate among critically ill patients.

Tocilizumab is a monoclonal antibody against interleukin-6 (IL-6). An alternative treatment for COVID-19 patients in patients at high risk of cytokine storm was suggested. The current evidence is still weak. A single-armed study on a small number of moderate to critically ill patients (*n* = 15) concluded that TCZ is effective in patients at high risk of cytokine storm [31]. Preventing the cytokine storm may be important for the treatment of COVID-19 infected patients. Due to high expression of ACE2 receptor in the alveolar type II cells and capillary endothelium, COVID-19 can stimulate a terrible cytokine storm in the lung, followed by oedema, dysfunction of the air exchange, ARDS and/or acute cardiac injury [60].

Corticosteroids’ role is widely reported in the management of COVID-19 [24,34,61]. Corticosteroid benefits in COVID-19 treatment were investigated in two observational retrospective studies, and both of them reported no association between adding steroids to standard treatment and patients’ outcome [2,20]. Thus, the current evidence indicates that the benefit of general use is inconclusive and is likely outweighed by adverse effects.

Disseminated intravascular coagulation causes the death of 71.4% of patients with COVID‐19 [62]. Tissue plasminogen activator production is associated with haemorrhagic fever, which could be caused by viruses as the dengue virus, to control fibrinolysis [63]. Besides, heparin has an inhibitory effect of herpes simplex virus [64]. tPA causes improvement in the oxygenation of COVID-19 patients and heparin improves their prognosis. However, their effect was weak, and more studies with a larger sample size are needed to evaluate their role clearly [22,35].

Being both highly pathogenic coronavirus with lung tropism, COVID-19 and SARS-CoV were found to bind to the same entry receptor (ACE2) with similar affinity. Furthermore, SARS-CoV polyclonal Antibodies inhibit COVID-19 spike glycoprotein (S)-mediated entry into cells [60]. The pilot clinical trial of MSCs transplantation to critically ill patients was found to be safe and successful. The study reported that in two days, all patients (*n* = 10) improved. MSCs have a powerful immunomodulatory ability that may have beneficial effects for preventing or attenuating the cytokine storm [11].

The efficacy of antiviral drugs namely lopinavir/ritonavir was assessed in three studies (two trials [12,13], one case series [28]) which reported conflicting results. Ye et al. concluded that the drugs have more evident therapeutic effect than pneumonia-associated adjuvant drugs alone [12]. Also, Wang et al. reported improvement of all (*n* = 4) patients [28]. On the other hand, the other clinical trial which was randomized and larger (*n* = 199) reported that treatment was stopped early in 13 patients (13.8%) because of adverse events and no benefit was observed with lopinavir/ritonavir treatment beyond standard care [13]. Antiviral treatment, including lopinavir/ritonavir may not be effective and warrants further verification in future study.

Remdesivir is an experimental drug with broad- spectrum antiviral agents. It was synthesized and developed by Gilead Sciences in 2017 as a treatment for ebola virus infection. Preclinical studies showed that it can inhibit coronaviruses, such as SARS-CoV and MERS-CoV replication [65]. Grein et al. study showed that severe cases with COVID-19, who were treated with compassionate use of remdesivir, were clinically improved (68% of them) [26]. Favipiravir is a pyrazine carboxamide derivative that has activity against RNA viruses. It inhibits RNA-dependent RNA polymerase enzymes and in turn inhibits viral transcription and replication. Favipiravir inhibits the replication of influenza and ebola. Favipiravir improves chest CT of patients with COVID-19 that may be crucial in the treatment of COVID-19 in the future [18].

Treatment of children infected with COVID-19 was described in three case series [23–25]. All these studies reported using the same guidelines of treatment (interferon, oseltamivir, antibiotics, interferon, respiratory support if needed, systemic corticosteroids and intravenous immunoglobulin). They reported favourable outcomes of treatment using the current medications. However, only one study conducted their study on severely ill children [24]. Thus, the current evidence of using a non-specific treatment in children especially severely ill is inadequate.

Being the only therapy for end-stage pulmonary fibrosis related to ARDS, lung transplantation has been considered as the ultimate rescue therapy for affected patients. One study included in this review reported that two out of three patients survived the surgery. The effectiveness of this option needs more evidence of efficacy. However, lung transplantation provided the final opportunity for these patients to avoid sure death, with the proper protection of transplant surgeons and medical staff [32].

The advantages of our scoping review are: (1) we included all the initial published studies that have been published on COVID-19, many of them are in Chinese language; (2) we provided a quality assess- ment of all the included studies, as they published in a very short time without efficient peer-reviewing; (3) our review presents new approaches that may be cru- cial in virology treatment in the future; (4) our review provides the main treatment options that will help the scientific society in their future research and practice. 

There are some limitations regarding our study: (1) most of the included studies have a low sample size; (2) most of the clinical trials are open-labelled and not randomized; (3) many studies lack many important clinical parameters; (4) several studies may be missed in our review.

## 5. Conclusions

The evidence regarding CQ and HCQ is still weak and needs further investigation especially with patients with severe comorbidities. Plasma transfusion and MSCs transplantation showed promising results in severely ill patients with no adverse effects; yet more studies are needed. The use of corticosteroids in treatment had no added benefits and may be harmful. No sufficient evidence to report on TCZ treatment. Treatment with lopinavir/ritonavir did not show higher effectiveness than the current guideline of non-specific treatments. Doctors may resort to lung transplantation to give patients the final opportunity with lung fibrosis, but effectiveness needs further studies to be verified.

## 6. Future research

Despite the extensive research on COVID-19, little is known about the virus. The exact pathology leading to death has not been elucidated. The variations between worldwide countries in terms of the infection and death rates are still unexplained. The questions about (1) whether the virus has genetic preferences, (2) whether the virus transmission and course are affected by temperature and (3) whether the disease course is influenced by prior vaccination history, are to be examined in future research. Recent studies found that ivermectin, baricitinib, baicalin, scutellarin, hesperetin, glycyrrhizin, nicotianamine and saikosaponins are potential compounds that might be promising in the prevention of viral entry or the inhibition of viral replication of COVID-19. Future research should translate the *in vivo* and *in vitro* results to the first-in-human trials of COVID-19 patients. Furthermore, there is a need for reliable, specific serological tests to detect COVID-19-specific antibodies and, therefore, determine the exact burden of the disease and determine the immune individuals who can return to work and restore the normal life.

## Abbreviations

ACE2: Angiotensin converting enzyme 2
ARDS: Acute respiratory distress syndrome
CD: Cluster of differentiation
COVID-19: Coronavirus disease 2019
CRP: C-reactive protein
CT: Computerized tomography
CXCR3: C-X-C motif chemokine receptor 3
CQ: Chloroquine
HCQ: Hydroxy chloroquine
HZ: Hazard ratio
IL: Interleukin
IQR: Interquartile range
MSCs: Mesenchymal stem cells
MERS: Middle East respiratory syndrome
PLT: Platelets
SARS: Severe acute respiratory syndrome
SFJDC: Shufeng Jiedu Capsule
TCZ: Tocilizumab
tPA: Tissue plasminogen activator
